# Supplementary material for: Lung Inflammasome Activation in SARS-CoV-2 Post-Mortem Biopsies
Source: Int J Mol Sci. 2022 Oct 27;23(21):13033. doi: 10.3390/ijms232113033 (PMC9659061; doi:10.3390/ijms232113033)
Supplement: Supplementary file 1 [file ijms-23-13033-s001.zip › ijms-1917655-supplementary.pdf]

Table S1. List of antibodies used in immunohistochemical staining.

Antibody	Type	Clone/Code	Dilution	Brand
**Anti-IL-18**	Polyclonal/Rabbit	EC9312	1:200	Elabscience
**Anti-IL-1β**	Polyclonal/Rabbit	A16288	1:50	ABclonal
**Anti-TNF-α**	Monoclonal/Rat	TA20	01:50	Santa Cruz
**Anti-CASP1**	Polyclonal/Rabbit	A0964	1:200	ABclonal
**Anti-CASP9**	Polyclonal/Rabbit	PAA627Hu01	1:100	Cloud-Clone
**Anti-GSDMD**	Polyclonal/Rabbit	A18281	1:100	ABclonal
**Anti-ASC**	Polyclonal/Rabbit	sc-33796	1:200	Santa Cruz
**Anti-NLRP-3/NALP**	Polyclonal/Goat	ab4207	1:100	Abcam
**Anti-NF-κB**	Polyclonal/Rabbit	ab7971	1:200	Abcam
**Anti-NOX4**	Polyclonal/Rabbit	Bs-6213R	1:100	Bioss
**Anti-TLR4**	Polyclonal/Rabbit	PA-23125	1:100	ThermoFisher
